# Identification and application of bioparts for plant synthetic biology

**DOI:** 10.1016/j.mocell.2025.100273

**Published:** 2025-09-01

**Authors:** Hyunjin Koo, Minah Jung, Sangwoo Lee, Sangjin Go, Yong-Min Kim

**Affiliations:** 1 Plant Systems Engineering Research Center, Korea Research Institute of Bioscience and Biotechnology (KRIBB), Daejeon 34141, Republic of Korea; 2 Department of Bioinformatics, KRIBB School of Bioscience, Korea University of Science and Technology (UST), Daejeon 34141, Republic of Korea; 3 Digital Biotech Innovation Center, Korea Research Institute of Bioscience and Biotechnology (KRIBB), Daejeon 34141, Republic of Korea

**Keywords:** Biopart, *cis*-regulatory elements (CREs), Plant synthetic biology, Promoter, Terminator

## Abstract

Plant synthetic biology is an emerging field that combines bioinformatics, computational gene circuit design, and plant science. It has the potential to be applied in various areas, including the production of pharmaceuticals, vaccines, biofuels, and various biomaterials, including plant natural products. This review highlights recent advancements in plant synthetic biology, particularly in the development and application of biological parts such as promoters and terminators, which play a crucial role in precise gene expression regulation. Furthermore, this review clarified the identification and utilization of bidirectional promoters, which are essential for gene pyramiding, and the significance of maintaining a balance between promoter and terminator combinations for the stability of transgene expression. Furthermore, large-scale identification of promoters using Assay for Transposase-Accessible Chromatin using sequencing and Self-Transcribing Active Regulatory Region sequencing, as well as deep-learning-based models for predicting promoter regions and their transcriptional activity, are discussed. This review provides insights into the identification and application of bioparts in plant synthetic biology to achieve efficient and precise gene regulation.

## INTRODUCTION

Plant synthetic biology integrates genome engineering, gene circuit design, and biological parts development to produce high-value biomaterials and address global challenges (Yang et al., 2022a). Recent advancements in genome editing (Yang et al., 2023), synthetic gene circuit design (Khan et al., 2024), and promoter engineering (Jores et al., 2021) have significantly expanded the molecular toolbox for precise and programmable gene expression in plants (Kong et al., 2025).

This field can be broadly divided into 2 main research areas: circuit design research and the development of biological parts. Circuit design research focuses on designing circuits to produce target biomaterials or proteins, and the development of biological parts involves the identification and development of biological components (such as promoters, terminators, signal peptides, untranslated resions (UTRs), and leader introns) for the precise regulation of gene expression in gene circuits. Identification and application of biological parts to achieve the goals of genetic engineering are critical in the design-build-test-learn cycle of plant synthetic biology (Wang and Demirer, 2023).

However, several key challenges remain unresolved, including the limited availability of well-characterized promoters and terminators, the inconsistent behavior of regulatory elements across different biological contexts, and the unpredictable nature of gene expression strength and spatiotemporal patterns. In addition, the combinatorial complexity of promoter-terminator interactions and lack of standardized design rules also hinder the portability and reliability of synthetic constructs across species and environments (Brooks et al., 2023, F de Felippes et al., 2020, Kummari et al., 2020, Tian et al., 2022).

While recent studies have focused on host and pathway optimization, comparatively little attention has been given to the regulatory elements required for robust and tunable gene expression. This review highlights current strategies for identifying and applying representative bioparts, especially promoters and terminators as well as large-scale discovery and deep-learning-based prediction of promoter activity.

## PROMOTERS AND TERMINATORS IN PLANTS

**Fig. 1:**
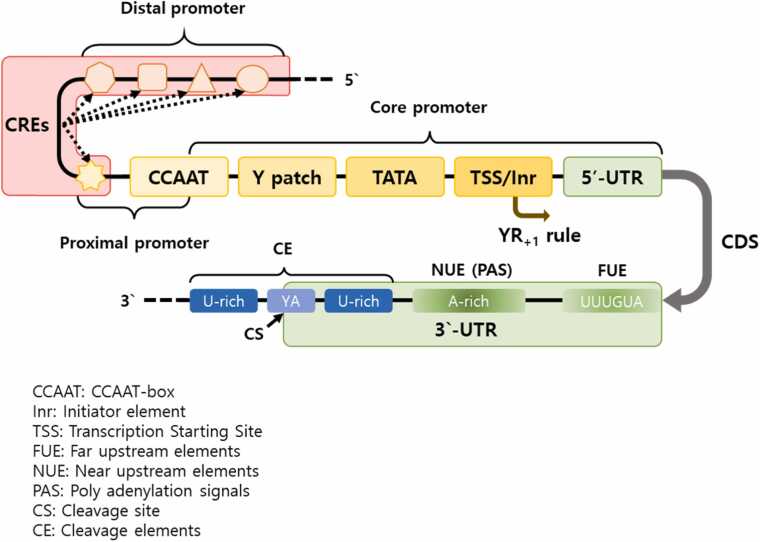
Schematic representation of a plant promoter and terminator. The promoter is composed of the core, proximal, and distal promoter regions. The borders of the plants FUE and NUE are diffused to highlight the variable size and shown as diffused colors. The following elements are indicated: CREs, *cis*-regulatory elements; CCAAT, CCAAT-box; TSS, transcription starting site; Inr, initiator element; FUE, far upstream elements; NUE, near upstream elements; PAS, polyadenylation signals; CS, cleavage site; CE, cleavage elements.

Promoters are the sequence of the 5′ upstream region of a gene that regulates the level of gene expression and are classified into constitutive promoters, tissue-specific promoters, and inducible promoters based on their expression patterns (Brooks et al., 2023, F de Felippes et al., 2020, Kummari et al., 2020, Tian et al., 2022). In plants, promoters are composed of core, proximal, and distal elements based on the function of the regulatory elements present in each region and their proximity to the transcriptional start site (TSS). The core promoter is composed of conserved, direction-sensitive motifs such as CCAAT-box, initiator element (Inr), TSS, and Y patch, while the Y patch is a plant–specific core promoter element (Fig. 1) (Jores et al., 2021, Yamamoto et al., 2007).

Securing promoters with varying levels of expression strength and specific gene expression patterns is essential for the bioengineering of complex plants (Brooks et al., 2023). To date, approximately 8,000 plant promoters have been identified through transcriptome and genome analysis and deposited in public databases, such as PlantPromDB (Shahmuradov and Solovyev, 2015), PlantPromoterDB (Hieno et al., 2014), and PlantCARE (Lescot et al., 2002). Among them, approximately 580 experimentally validated promoter regions are provided in PlantPromDB for further applications (Hieno et al., 2014).

Terminators, located downstream of coding sequence, ensure proper 3′ end processing, polyadenylation, and transcript stability (De Felippes and Waterhouse, 2023, Hunt, 2008, Hunt, 1994, Rothnie, 1996). They consist of 3 major *cis*-elements, which are the far upstream elements, the near upstream element (NUE), and the cleavage site (Fig. 1). While NUEs in mammals typically contain a conserved AAUAAA motif, plant terminators often diverge in sequence and structure, with only a minority containing the canonical polyadenylation signal (De Felippes and Waterhouse, 2023). The variability in plant terminator elements contributes to inconsistent transcript termination and mRNA stability.

## APPLICATIONS FOR PROMOTERS AND TERMINATORS IN PLANT SYNTHETIC BIOLOGY

Precise control of gene expression through well-designed promoters and terminators is essential for constructing efficient genetic circuits in plant synthetic biology. Plant transformation using tissue culture is a rate-limiting step in plant synthetic biology that introduces new gene circuits or genome editing-based metabolic pathway reconstruction (Lowe et al., 2018, Lowe et al., 2016, Maher et al., 2020).

Recent studies demonstrated that the use of morphogenic regulators such as Baby Boom and Wuschel2, driven by tissue-specific or inducible promoters, enhances transformation efficiency while avoiding sterility or abnormal phenotypes caused by constitutive expression (Lowe et al., 2016). For example, the embryo-specific phospholipid transfer protein (Zm-PLTPpro) promoter and auxin-inducible AXIG1 promoter enabled efficient maize regeneration without developmental defects (Lowe et al., 2018).

To regulate or reconstruct plant metabolic pathways, introducing and expressing partial or complete biosynthetic genes from native to host species is a key strategy. In golden rice, 3 carotenoid biosynthesis genes were regulated by endosperm-specific glutelin (Gt1) and CaMV 35S promoters (Al-Babili and Beyer, 2005). Replacing 35S with an endosperm-specific promoter significantly enhanced carotenoid levels, highlighting the role of promoter selection (Al-Babili and Beyer, 2005). Similarly, purple rice was engineered using 8 endosperm-specific promoters to drive anthocyanin biosynthetic genes individually, showcasing promoter diversity as a valuable tool in plant metabolic engineering (Zhu et al., 2017).

In addition to promoters, terminators also play a critical role in determining gene expression outcomes in plant synthetic biology. Recent studies have demonstrated that terminator selection significantly affects transcript accumulation, readthrough suppression, and resistance to gene silencing. For instance, native plant-derived terminators such as *tHSP18* and *tMIR* frequently outperform exogenous elements like *tNos*, resulting in higher and more stable transgene expression. Moreover, strategies such as dual-terminator combinations and the inclusion of matrix attachment regions have been shown to further enhance expression levels and reduce variability. These findings underscore the importance of co-optimizing promoter-terminator pairs when engineering robust synthetic circuits in plants (Brooks et al., 2023).

## BIDIRECTIONAL PROMOTERS FOR GENE PYRAMIDING

Various bidirectional promoters have been identified in different plant species and applied to diverse area of plant biotechnology (Table 1). However, repetitive use of a single promoter may cause transcriptional gene silencing in transgenic plants (Ajikumar et al., 2010, De Wilde et al., 2000). Several methods, such as self-cleaving 2A peptide and bidirectional promoter, have been developed to avoid this unexpected effect. Bidirectional promoters are defined as the intergenic region between gene pairs located on opposite strands of DNA with TSS in opposing directions. Bidirectional gene pairs tend to be highly coregulated and function in similar biological processes in eukaryotic genomes, and intergenic regions of bidirectional gene pairs are defined as approximately 1 kb apart (Wang et al., 2009). Bidirectional promoter activity in plant was first demonstrated using synthetic constructs in *Arabidopsis thaliana* (Xie et al., 2001), which included various promoter element including 35S, SAG12, and OPR1. Three native bidirectional promoters were reported in *A. thaliana*, rice, and hot pepper (Mitra et al., 2009, Shin et al., 2003, Singh et al., 2009). Then, genome-wide identification of functional bidirectional promoters was carried out in *A. thaliana*, rice, *Populus*, and maize (Dhadi et al., 2009, Kourmpetli et al., 2013, Liu et al., 2014, Wang et al., 2009). In total, 27 from 2,471 bidirectional gene pairs in *A. thaliana* were identified (Wang et al., 2009). Bidirectional promoters are applied to construct CRISPR/Cas9 systems (Ren et al., 2019) and tissue-specific synthetic promoters (Bai et al., 2020).

**Table 1:** List of bidirectional promoters

Promoter name	Type	Source	Species tested	Reference
pGLbd1	Synthetic	P35Smini::CaMV35S	*Arabidopsis thaliana*	Xie et al. (2001)
pGLbd3		SAGmini::CaMV35S		
pGLbd4		P35Smini::PCISV		
pGLbd5 and 6		P35Smini::OPR1		
CaTin1-2 promoter	Native	CaTin1-2 and CaTin1 intergenic region	*Capsicum annuum*	Shin et al. (2003)
Bidirectional promoter in rice	Native	OCPI2 and OCPI1 intergenic region	*Oryza sativa*	Singh et al. (2009)
Bidirectional promoters in rice, *Arabidopsis* and *Populus*	Native	Intergenic regions within gene pairs of rice, *Arabidopsis*, and *Populus*	*O. sativa, A. thaliana, Populus trichocarpa*	Dhadi et al. (2009)
Bidirectional promoters in *Arabidopsis*	Native	Intergenic regions between the *Arabidopsis* gene pairs	*A. thaliana*	Wang et al. (2009)
Bidirectional promoter in *Arabidopsis*	Native	cab1 and cab2 intergenic region	*A. thaliana*	Mitra et al. (2009)
Bidirectional promoters in *Arabidopsis*	Native	Intergenic regions between the *Arabidopsis* gene pairs	*A. thaliana*	Kourmpetli et al. (2013)
BiP1	Native	LOC_Os02g42314 and LOS_Os02g42320 intergenic region	*O. sativa*	Wang et al. (2016)
BiGSSP2	Synthetic	POsrbcs-550::POsrbs-62::OsAct1 intron	Transgenic rice	Bai et al. (2020)
BiGSSP3	Synthetic	POsrbcs-550::4×GEAT::POsrbcs-62::OsAct1 intron		
BiGSSP6	Synthetic	POsrbcs-550::PD540-544::OsAct1 intron		
BiGSSP7	Synthetic	OsTub6 intron::POsrbcs-550::PD540-544::OsAct1 intron		
Pmas201	Native	*mas1'-2'* bidirectional promoter from *Rhizobium radiobacter*	*Tanacetum cinerariifolium*	Shinoyama et al. (2024)
P1301	Synthetic	Pmec::TAM	*Nicotiana tabacum*	Chaturvedi et al. (2006)
pd35GR	Synthetic	C1::E2::E2::E1::E1::C1; C1(CaMV 35S core promoter), E2(35S promoter enhancer fragment), E1(35S promoter enhancer fragment)	*Vitis vinifera, N. tabacum*	Li et al. (2004)
FsFfCBD	Synthetic	FMV-Sgt::FMV-Flt::CaMV35S	*N. tabacum*	Patro et al. (2013)

## GENE HARMONY MODEL FOR APPLICATION OF COMBINED PROMOTER-TERMINATOR COMBINATION FOR PLANT SYNTHETIC BIOLOGY

To achieve the ends of genetic engineering, precise spatiotemporal regulation of gene expression is essential for plant synthetic biology. Previous studies demonstrated that terminators play crucial roles in the highest protection against gene silencing by inhibiting transgene-derived sRNA production and contributing the most to the final levels of gene (Dadami et al., 2014, Dadami et al., 2013, De Felippes and Waterhouse, 2020, Luo and Chen, 2007, Nicholson and Srivastava, 2009). To minimize readthrough transcription, genes have evolved mechanisms that balance transcription initiation and termination (De Felippes and Waterhouse, 2023).

Based on this, recent studies suggest the gene harmony model balancing between promoter and terminator to enhance the stability of transgenes against RNA silencing (De Felippes and Waterhouse, 2020, De Felippes and Waterhouse, 2023). In addition to balancing transcription initiation and termination under normal conditions, environmental conditions also impact promoter and terminator function. Stress-responsive promoters contain *cis*-regulatory elements (CREs) such as ABRE, DRE, and W-box, which are recognized by transcription factors activated under specific signaling pathways (Eulgem and Somssich, 2007, Yamaguchi-Shinozaki and Shinozaki, 2005, Zou et al., 2011). Concurrently, stress conditions can influence transcription termination efficiency and alternative polyadenylation, often resulting in transcripts with shorter 3′ UTRs. These shorter UTRs enhance transcript stability by avoiding sequences that trigger mRNA degradation (Vilborg et al., 2017, Zhou and Li, 2023). These findings highlight the need to optimize promoter-terminator combinations for stress-resilient synthetic circuits.

Gene expression has been shown to vary by over 300-fold depending on the specific promoter-terminator combination, highlighting the importance of pairing optimization (Pfotenhauer et al., 2022, Tian et al., 2022). Similarly, the use of tandem terminators in conjunction with geminiviral vectors can enhance transgene expression by more than 150-fold compared to conventional terminators such as *tNos*, and the combination of multiple plant-derived terminators has been reported to result in more than a 60-fold increase in protein expression, emphasizing that terminators function as active regulators of gene expression rather than passive elements (Rizzo et al., 2023).

## BENCHMARKING PROMOTERS AND TERMINATORS

Despite the identification of over 8,000 plant promoters through multiomics analyses, only a limited number have undergone functional validation in planta (Brooks et al., 2023). To support precise genetic and metabolic engineering in plant synthetic biology, it is essential to validate promoters and terminators using quantitative, robust methods. Key parameters for evaluation include temporal and spatial expression patterns, environmental responses, and cross-species variation. Functional validation is typically performed using transient or stable expression systems. Transient expressions, such as protoplast transfection, leaf agro-infiltration, or hairy root transformation, enable rapid and flexible testing across plant tissues. In contrast, stable transformation provides more definitive insights into the spatiotemporal dynamics and long-term performance of regulatory elements, though it is labor-intensive.

**Fig. 2:**
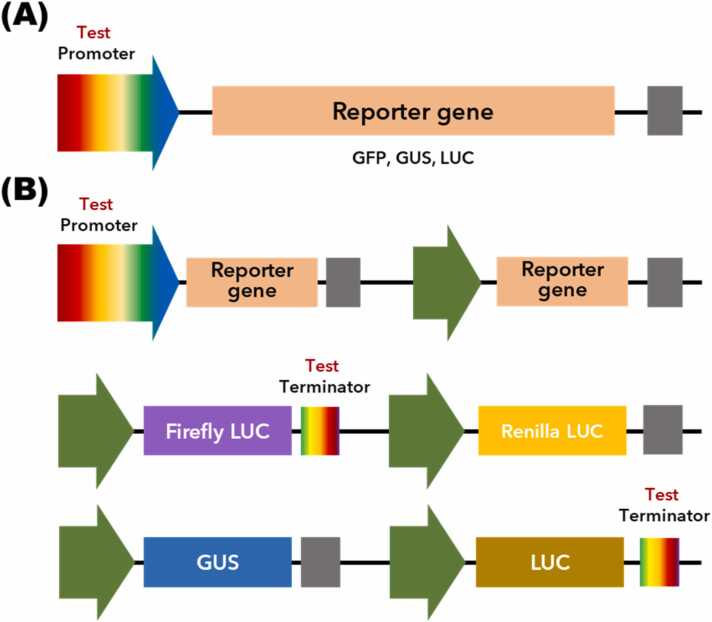
Schematic diagram of constructs used for the single and dual reporter systems. (A) Constructs a single-reporter system for investigating transcriptional activity of promoters. (B) Constructs a dual reporter system for investigating transcriptional activity of promoters and terminators. Green arrows represent 35S promoter. The following elements are indicated: GUS, β-glucuronidase; GFP, green fluorescent protein; LUC, luciferase.

Single-reporter systems, employing genes such as β-glucuronidase (GUS) (Han et al., 2015), green fluorescent protein (GFP) (Belcher et al., 2020), and luciferase (LUC) (Cai et al., 2020), remain widely used in assay (Fig. 2A). These systems offer quantitative insights but are limited by factor such as autofluorescence, enzymatic accumulation over time, or variability in expression due to transgene integration site effects. Reverse transcription quantitative polymerase chain reaction and droplet digital polymerase chain reaction complement these systems by enabling precise transcript quantification. A dual reporter system improves normalization of reporter gene expression by using a weak promoter-terminator combination as an internal control (Brooks et al., 2023, Cai et al., 2020, Tian et al., 2022). This setup enhances precision by minimizing positional effects in stable lines. Several dual reporter systems have been developed for quantitative promoter and terminator evaluation in plants (Fig. 2B).

## RESEARCH ON SYNTHETIC PROMOTERS

Synthetic promoters, composed of core promoter elements and CREs, enable precise spatiotemporal control of gene expression and offer improved predictability over native promoters (Liu and Stewart, 2016, O’Malley et al., 2016). Although CRE identification in plants remains challenging due to context dependency, recent approaches have advanced their systematic characterization. In crops, synthetic promoters constructed from soybean-derived CREs showed strong and specific inducibility in nematode-infected root tissues, demonstrating their potential for targeted transgene expression in agriculture (Sultana et al., 2022). Drought-inducible promoters, designed computationally, have also been applied in transgenic poplar (Yang et al., 2021, Yang et al., 2022b). To construct and evaluate synthetic promoter libraries, studies have employed combinatorial assembly of CREs and core promoters, as well as genome-wide enhancer screening via Self-Transcribing Active Regulatory Region sequencing (STARR-seq). These libraries are typically screened using transient assays such as LUC or GUS reporters, enabling scalable and quantitative assessment of promoter activity (Cai et al., 2020, Jores et al., 2020).

## LARGE-SCALE IDENTIFICATION OF PROMOTERS

Precise transcriptional regulation is foundational for designing robust gene circuits in plant synthetic biology. While traditional promoter discovery has relied on sequence-based analyses of upstream regions containing conserved motifs such the TATA box, CCAAT-box, Inr, TSS, and Y patch (Civáň and Švec, 2009, de Medeiros Oliveira et al., 2021, Kaur et al., 2017), these approaches are limited in their ability to capture tissue-specific or condition-dependent promoter activity in complex plant genomes.

**Fig. 3:**
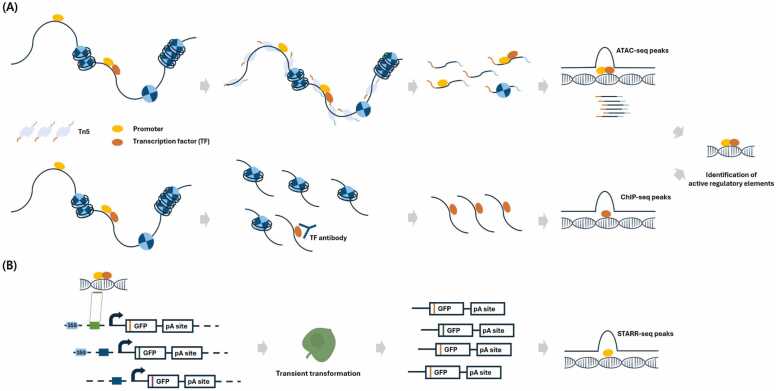
Overview of genome-wide identification of promoters. (A) ATAC-seq reveals open chromatin regions, while ChIP-seq identifies transcription factor binding sites, further narrowing down promoters with active transcriptional regulation. (B) Validation of candidate promoter activity using STARR-seq. The following elements are indicated: GFP, green fluorescent protein; pA site, polyA site.

To address this, Assay for Transposase-Accessible Chromatin using sequencing (ATAC-seq) has emerged as a powerful genome-wide method to identify accessible chromatin regions, which are enriched in active regulatory elements, including promoters or regulatory elements (Chen et al., 2024, Maher et al., 2018, Starks et al., 2019). However, while ATAC-seq sheds light on accessible regions of the genome, it does not provide information on whether specific transcription factors or histone modifications are interacting with these regions. To address this limitation, Chromatin Immunoprecipitation Sequencing (ChIP-seq) can be employed to identify transcription factor binding sites and histone modifications known to regulate gene expression (Hu et al., 2010). Previous studies, such as one involving the ASR1 transcription factor in *Solanum lycopersicum* under drought stress, have demonstrated the application of ChIP-seq for uncovering promoter activity (Ricardi et al., 2014). This method validates whether the identified open chromatin regions contain active promoters by providing evidence of transcription factor bindings and regulatory modifications, allowing for the identification of functionally relevant promoters (Fig. 3A).

In addition, STARR-seq enables functional screening of promoters and enhancers by linking their activity to reporter gene expression. In STARR-seq, candidate promoters are inserted upstream of the GFP gene with or without the enhancer of 35S promoters in a reporter construct, and their transcriptional activity is directly measured by the strength of the transcribed reporter RNA (Fig. 3B) (Arnold et al., 2013, Kim et al., 2021). This enables simultaneous assessment of promoter and enhancer activity. Recently, it has been applied to crops such as rice, tobacco, and maize, leading to the discovery of numerous functional enhancers, promoters, and CREs (Jores et al., 2020, Ricci et al., 2019, Sun et al., 2019).

While conventional promoter screening approaches such as LUC- or GUS-based transient assays remain widely used for their simplicity and tissue-specific resolution, STARR-seq offers a scalable, quantitative method to screen enhancer and promoter activity genome-wide. Although its application in plants is still emerging, recent studies in tobacco have demonstrated that STARR-seq enables the identification of CREs and provides higher-throughput capabilities than traditional reporter assays (Jores et al., 2020). Thus, STARR-seq complements conventional methods by enabling broader discovery and functional classification of regulatory elements. The integration of these methods provides a comprehensive framework for identifying and validating promoters, accelerating the development of reliable plant synthetic biology tools.

## DEEP-LEARNING-BASED PREDICTION OF PROMOTERS AND THEIR TRANSCRIPTIONAL ACTIVITY

Sequence-based prediction of promoters and terminators in plants is challenging due to genome complexity, large genome size, polyploidy, and whole-genome duplications. Deep learning models have recently improved the prediction of promoter sequence and activity. Convolutional neural networks (CNN), long short-term memory (LSTM), and Bidirectional Encoder Representations from Transformers (BERT) are widely used to identify promoter patterns in DNA sequences (Zeng et al., 2021) (Table 2). Promoter prediction research focuses on 3 areas: promoter region prediction, TATA/TATA-less promoter identification, and promoter type characterization.

**Table 2:** List of deep-learning-based tools for promoter prediction

Description	Tool	Algorithm	Species	Database	Sequence length (bp)	Reference
Characterization of promoter and nonpromoter regions	CNNProm	CNN	*E*. *coli* *B*. *subtilis*	EPD	81	Umarov and Solovyev (2017)
	Cr-Prom	CNN	*O. sativa* *A. thaliana* *Z*. *mays*	PlantProm RAP-DB	251	Shujaat et al. (2021)
	CapsProm	CapsNet	*E. coli* *B. subtilis*	Benchmark dataset (CNNProm) DBTBS RegulonDB	81	Moraes et al. (2022)
	iProm-Zea	CNN	*Z. mays* *A. thaliana* *Oryza* *Glycine* *P*. *sativum*	EPD PlantProm	251	Kim et al. (2022)
Prediction for TATA and TATA-less promoters	CNNProm	CNN	*H*. *sapiens* *M*. *musculus* *A. thaliana*	Ensembl DBTSS EPD	251	Umarov and Solovyev (2017)
	DeePromoter	CNN, BiLSTM	*H. sapiens* *M. musculus*	EPD	300	Oubounyt et al. (2019)
	CapsProm	CapsNet	*H. sapiens* *M. musculus* *A. thaliana*	Benchmark dataset (CNNProm)	251	Moraes et al. (2022)
Promoter prediction from TATA, TATA-less, and mixed datasets	DeeReCT-PromID	CNN	*H. sapiens*	EPD	600	Umarov et al. (2019)
	Depicter	CapsNet	*H. sapiens* *M. musculus* *Drosophila melanogaster* *A. thaliana*	EPD EID FlyBase	300	Zhu et al. (2021)
	DeeProPre	CNN, BiLSTM	*D. melanogaster* *M. musculus*	Benchmark dataset (Deciper)	300	Ma et al. (2022)
Classification for types of Sigma promoters	iPromoter-BnCNN	CNN	*E.coli*	RegulonDB	81	Amin et al. (2020)
Identification of promoters and prediction of their strength	BERT-Promoter	BERT, ML	*E.coli*	Benchmark datset (iPSW(2L)-PseKNC)	81	Le et al. (2022)
	msBERT-promoter	BERT	*E.coli*	Benchmark datset (iPSW(2L)-PseKNC)	81	Li et al. (2024)

BERT, Bidirectional Encoder Representations from Transformers; CapsNet, Capsule Network; CNN, convolutional neural networks; DBTBS, a database of *Bacillus subtilis* promoters and transcription factors; DBTSS, Database of Transcriptional Start Sites; EID, Exon-Intron Database; EPD, Eukaryotic Promoter Database.

CNN-based models have demonstrated remarkable results. CNNProm predicted TATA and TATA-less promoters in *A. thaliana* by detecting transcription initiation motifs (Umarov and Solovyev, 2017). iProm-Zea is a 2-layer CNN model for *Zea mays* promoter prediction, where the first layer detects promoter regions and the second characterizes promoter types (Kim et al., 2022). Additionally, iPromoter-BnCNN utilized a customized CNN model to classify 6 types of sigma promoters in *Escherichia coli* by capturing local nucleotide features (Amin et al., 2020). To improve precision, hybrid CNN-LSTM has been developed, where CNN detects sequence motifs and LSTM captures long-distance dependencies (Alharbi and Rashid, 2022, Kaur et al., 2022). DeePromoter used a CNN model for detecting promoters in humans and mice and bidirectional LSTM layers for long-distance dependencies (Oubounyt et al., 2019). Similarly, DeeProPre combined CNN and bidirectional long short-term memory (BiLSTM) to enhance prediction precision by considering both local features and sequence positions for promoter classification for *Drosophila* and mice (Ma et al., 2022).

Advanced models such as BERT and Graph Neural Network have been applied to promoter prediction. BERT-Promoter (Le et al., 2022) identifies promoter regions and predicts transcriptional strength of core promoters for *E. coli* using SHAP feature selection to reduce sequence redundancy. msBERT-Promoter enhances promoter prediction using a multiscale ensemble approach to classify promoters and predict their strength, incorporating tokenization strategies and soft voting, which aggregates probabilities from multiple models (Li et al., 2024). GraphPro (Zhang et al., 2024), a Graph Neural Network-based model, identifies promoters across multiple species by constructing graphs where each node represents a DNA sequence and edges represent DNA pairing potentials. Although applications of advanced models to plant genomes are limited, the success in other organisms suggests potential for plant systems. With deep learning advances, application to promoter prediction in plants is expected to broaden, enabling more accurate identification of candidate promoters and advancing plant genomic research.

## Author Contributions

**Yong-Min Kim:** Writing – review & editing, Supervision. **Hyunjin Koo:** Writing – original draft, Conceptualization. **Minah Jung:** Writing – original draft. **Sangwoo Lee:** Writing – original draft. **Sangjin Go:** Visualization, Conceptualization.

## Declaration of Competing Interests

The authors declare that they have no known competing financial interests or personal relationships that could have appeared to influence the work reported in this paper.
